# The Effect of Chronic Prostatitis/Chronic Pelvic Pain Syndrome (CP/CPPS) on Erectile Function: A Systematic Review and Meta-Analysis

**DOI:** 10.1371/journal.pone.0141447

**Published:** 2015-10-28

**Authors:** Xiang Chen, ZhiRui Zhou, XiaoChun Qiu, Bin Wang, JiCan Dai

**Affiliations:** 1 Department of Urology, Ren Ji Hospital, School of Medicine, Shanghai Jiao Tong University, Shanghai, China; 2 Department of Radiation Oncology, Fudan University Shanghai Cancer Center, Shanghai, China; 3 Department of Oncology, Shanghai Medical College, Fudan University, Shanghai, China; 4 Library of Shanghai Jiao Tong University, School of Medicine, Shanghai, China; 5 Department of Andrology, Dongzhimen Hospital, Beijing University of Chinese Medicine, Beijing, China; The James Cook University Hospital, UNITED KINGDOM

## Abstract

**Background:**

High prevalence of erectile dysfunction (ED) has been observed in patients with chronic prostatitis/chronic pelvic pain syndrome (CP/CPPS). However, whether or not CP/CPPS is a risk factor of ED remains unknown and controversial. Therefore, we conducted this systematic review and meta-analysis to evaluate the relationship between CP/CPPS and ED.

**Methods:**

PubMed, Embase, Web of Science, and The Cochrane Library were searched up to November 11, 2014 to identify studies reporting the association between CP/CPPS and ED. Case–control, cohort and cross-sectional studies were included. Quality of the included studies was assessed. The odds ratio of ED and the mean difference of five-item International Index of Erectile Function (IIEF-5) score were pooled using a random effects model. Subgroup analysis and sensitivity analyses were performed.

**Results:**

Three cross-sectional studies, two case–control studies, and four retrospective studies with 31,956 participants were included to calculate the pooled odds ratio of ED, and two studies with 1499 participants were included to calculate the pooled mean difference of IIEF-5 scores. A strong correlation was found between CP/CPPS and ED (pooled odds ratio: 3.02, 95% CI: 2.18–4.17, *P* < 0.01), with heterogeneity across studies (*I* ^*2*^ = 65%; *P* < 0.01). A significant decrease in the IIFE-5 score was observed in the CP/CPPS group (pooled mean difference: −4.54, 95% CI: −5.11–−3.98; *P* < 0.01).

**Conclusion:**

Our study indicates that patients with CP/CPPS have an increased risk of suffering from ED. Assessment of erectile function is necessary for the therapy of patients with CP/CPPS. Further evidence is necessary to confirm the relationship between CP/CPPS and ED.

## Introduction

Erectile dysfunction (ED) is defined as the persistent inability to attain and maintain an erection sufficient to permit satisfactory sexual performance [1]. It is a major male sexual dysfunction with a prevalence of 2%–20% in men younger than 50 years old and 20–40% in men aged 60–69 years old [2]. Prostatitis is a common urological disease that impairs the quality of life of men in many aspects. According to the National Institutes of Health (NIH), prostatitis may be classified into four categories [3], among which category III is chronic prostatitis/chronic pelvic pain syndrome (CP/CPPS). A similar definition for chronic pelvic pain syndrome and prostate pain syndrome is recommended by International Association for the Study of Pain, and is also widely used [4]. The National Institutes of Health chronic prostatitis symptom index (NIH-CPSI) is a valid tool widely used to assess CP/CPPS in clinical practice [5]. The prevalence of CP/CPPS assessed using NIH-CPSI is about 8%–10% [6,7].

Studies noted a high prevalence of ED in men with CP/CPPS [8], but most of these studies only enrolled CP/CPPS patients with no control group. UPOINT is an effective phenotype system directing the multimodal therapy of CP/CPPS patients. The initial UPOINT system contains six domains: urinary, psychosocial, organ specific, infection, neurologic/systemic, and tenderness [9]. Adding a sexual domain to this system can improve the correlation with symptom severity in CP/CPPS patients [10,11]; however, results regarding this point are controversial [12,13].

Evidence suggests a link between CP/CPPS and ED, but the underlying mechanisms are unclear. CP/CPPS is associated with increased risk factors of ED, which includes arterial stiffness and endothelial dysfunction [14]. However, psychological factors may play a key role in the genesis of ED in CP/CPPS patients. CP/CPPS patients suffer from considerable stress, depression, and anxiety [15]. These psychological disorders, along with pain symptoms and voiding dysfunction, may decrease sexual activity and erectile function [16]. Endocrine and neurologic factors may also be involved in the pathogenesis of ED in CP/CPPS individuals [8].

Whether or not CP/CPPS is a risk factor of ED remains to be clarified. Therefore, we conducted a systematic review and meta-analysis of published case–control, cohort and cross-sectional studies to evaluate the association between CP/CPPS and ED in adult men. We estimated the odds ratio (OR) of ED and the mean difference of five-item International Index of Erectile Function (IIEF-5) scores between men with CP/CPPS and controls. Subgroup analysis was also performed.

## Methods

A study protocol was developed for this review and was registered in PROSPERO International prospective register of systematic reviews (ID: CRD42014015113,http://www.crd.york.ac.uk/PROSPERO/display_record.asp?ID=CRD42014015113#.VQ7eKdLWI4I). We reported this systematic review in accordance with Preferred Reporting Items for Systematic Reviews and Meta-Analyses (i.e., the PRISMA statement) [17].

### Literature search

PubMed, Embase, The Cochrane Library, and Web of Science were systematically searched from inception of the databases to Nov 11, 2014. Combination of medical subject headings, terms, and corresponding free text words related to “chronic prostatitis,” “chronic pelvic pain” and “erectile dysfunction” were used in the electronic search. Details of the search strategy are available in Document A in S1 Appendix. The bibliographies of the pertinent articles as well as reviews were manually searched for additional records. No language restriction was applied. The search strategy was developed by XC and XCQ.

### Study selection

Included studies should meet the following criteria: (1) CP/CPPS and ED were independently defined and reported in any criteria but not in a mixed criterion, e.g., prostatic disease and sexual dysfunction; (2) sufficient data were provided to calculate the odds ratio of ED or the mean difference of IIEF-5 scores; (3) for multiple reports from the same population, only the most recent or complete publications was included; and (4) case–control, cohort (retrospective or prospective), or cross-sectional design were employed. We defined a cohort study as retrospective if ED occurred before data collection. Meanwhile, we defined a cohort study as prospective if ED did not occur at the beginning of the study and a follow-up visit was designed.

### Data extraction

The titles and abstracts of existing studies were initially independently screened by two reviewers (XC and ZRZ). Full texts of potentially relevant papers were reviewed later. The reference lists of the relevant studies and reviews were manually searched. Personal contact was tried to obtain research information if necessary. Discrepancies were resolved through discussion with a third reviewer (JCD). Data extracted included first author, publication year, study design, country, definition of CP/CPPS and ED, control selection, sample size, number of patients with ED in each group, age, and IIEF-5 scores, among others. These data were checked by a third reviewer (BW).

### Quality assessment

The Newcastle–Ottawa Scale (NOS) developed by Wells et al was used to assess case–control and cohort studies [18]. The 11-item table developed by Rostom et al was used to assess cross-sectional studies [19]. Two of the authors (XC and ZRZ) performed the quality assessment procedure respectively. Disagreements were solved through a discussion with a third reviewer (JCD).

### Data synthesis and analysis

The Q statistic and *I* ^*2*^ index were calculated to test for heterogeneity. Quantitative meta-analyses were performed using a random-effects model because these studies were conducted in various populations and different designs were employed. The odds ratio of ED and the mean difference of IIEF-5 scores were measured. The definitions of ED and CP/CPPS were based on the descriptions provided in the included studies. If an included study reported two definitions for ED and CP/CPPS, we chose IIEF-5 reported ED and NIH criteria of CP/CPPS for the overall data synthesis. Subgroup analysis was conducted to investigate the possible sources of heterogeneity. To estimate the consistency of the overall effect, cumulative meta-analysis sorted by publication year was performed. We repeated the meta-analysis in a different model and we conducted sensitivity analysis by omitting studies one by one to assess whether or not the pooled results were markedly affected by any single study. Publication bias was evaluated by performing Peters’ test [20,21]. Statistical analysis was conducted using Review Manager (version 5.3, The Nordic Cochrane Centre, The Cochrane Collaboration, 2012, Copenhagen), Stata (version 13.0, College Station, Texas, USA) and metafor package of R (version 3.2.2, The R Foundation for Statistical Computing, Vienna, Austria).

## Results

**Fig 1:**
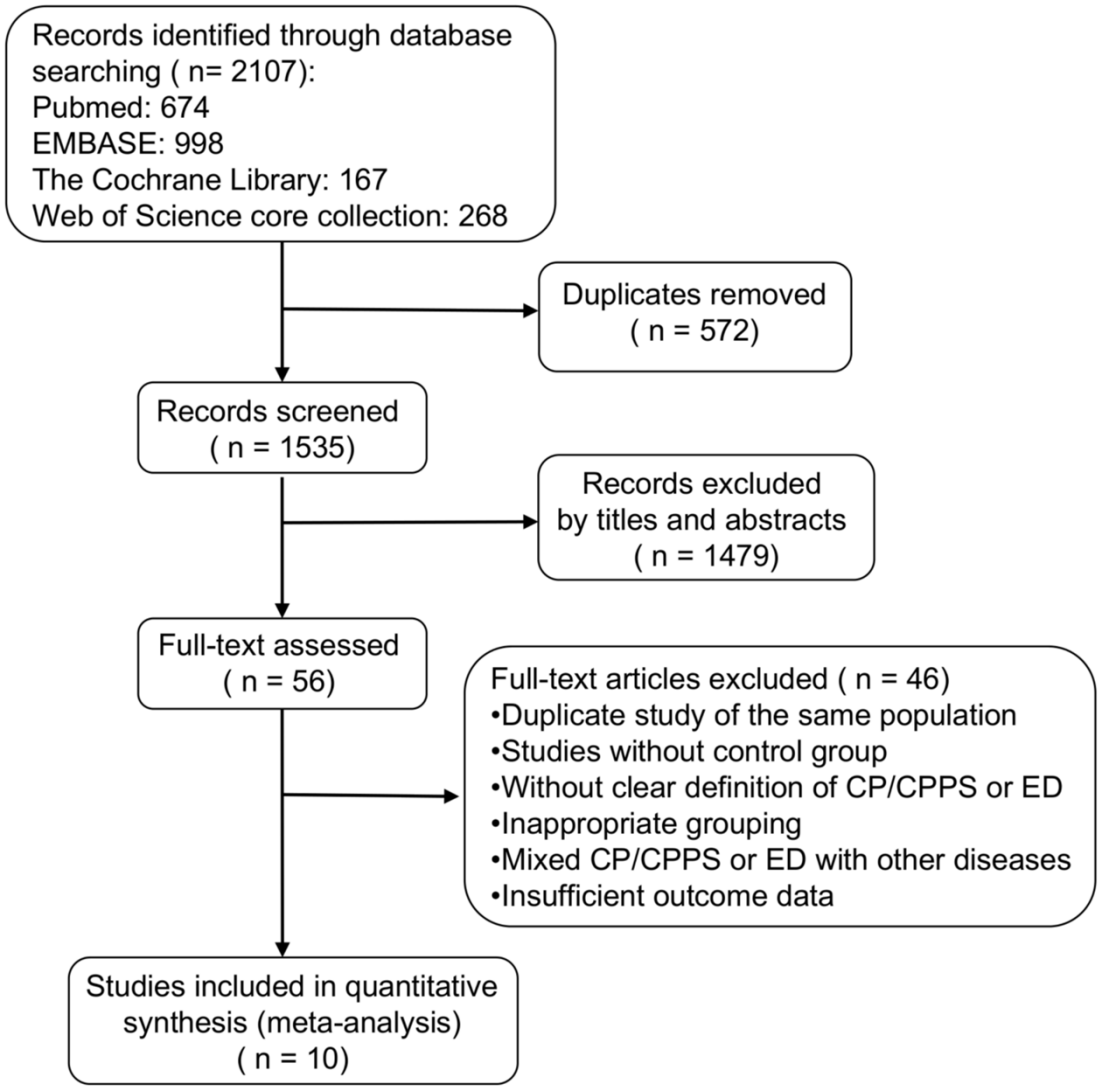
Flow diagram of literature search and study selection. CP/CPPS = chronic prostatitis/chronic pelvic pain syndrome, ED = erectile dysfunction.

A total of 2107 relevant records were identified by literature search. After screening the titles and abstracts, 56 were left for full text assessment. Finally 10 studies were included in the systematic review and meta-analysis. Nine of them were included in the quantitative analysis to calculate the OR of ED, and two were included to calculate the mean difference of IIEF-5 scores. The study selection process is shown in Fig 1. Full-text excluded articles and reasons for exclusion are available in Document B in S1 Appendix.

### Characteristics of the included studies

We included three cross-sectional studies [22–24], two case–control studies [25,26], and four retrospective cohort studies [27–30] with a total sample size of 31,956 participants (10,371 in cross-sectional study, 19,870 in case–control study, and 1715 in retrospective cohort study) for reporting the OR of ED. Two studies with a sample size of 1499 participants were included for reporting the mean difference of IIEF-5 scores [29,31]. Detailed characteristics of the studies are listed in Table 1. Most of the included studies were published in English except for one (Fan, 2012, in Chinese) [24]. Most of the individual studies collected data via interview and questionnaire survey, but one study (Chung, 2012) extracted data from a health insurance database [25]. The diagnosis criteria of ED and CP/CPPS varied across studies. One study (Hao, 2011) reported results in two criteria of ED and CP/CPPS [22]; two studies reported results in the CP subtype of NIH criteria [29,30].

**Table 1:** Characteristics of included studies.

Study, publication year	Country	Age(mean or range), yr	Sample size	Population	Data source	CP/CPPS criteria	ED criteria	Control selection	Quality score
**Cross-sectional study**									
**Tan, 2002**	Singapore	41.68, 21–70	1087	Community based	Questionnaire survey	Pain or discomfort in the perineum, testicles, tip of penis or suprapubic region, associated with micturition	IIEF-5 score ≤21	N/A	8
**Rosen, 2009**	USA	30–79	2301	Community based, Boston Area Community Health survey	Interview	Perineal and/or ejaculatory pain and CPSI Pain score ≥4	IIEF-5 score ≤16	N/A	5
**Hao, 2011**	China	39.54, 22–60	7372	Community based, multi-regional	Questionnaire survey	CP like symptoms: complained of perineal or ejaculatory pain or discomfort and CPSI Pain score ≥4. CP: symptoms or laboratory test of prostatic secretion (white blood cell ≥10/high power objective) ≥3 months	Self-report: inability to sustain or achieve an erection sufficient for satisfactory intercourse. IIEF-5 report: IIEF-5 score ≤21	N/A	6
**Fan, 2012**	China	40–80	698	Community based, multi-regional	Interview with questionnaire survey	Medical history of CPPS	IIEF-5 score ≤21	N/A	7
**Case-control study**									
**Elbendary, 2009**	Egypt	20–40	434 cases, 272 controls	Hospital based	Interview with questionnaire survey	Medical history of CPPS	IIEF-5 score ≤21, patients with history suggesting psychogenic ED were excluded	Potent, healthy volunteers	6
**Chung, 2012**	Taiwan, China	51.9	3194 cases, 15970 controls	Multi-hospital based	Longitudinal Health Insurance Database 2000	Three month history of genitourinary pain and an absence of other lower urinary tract pathologies. Diagnosed with CP/CPPS (ICD-9CM code 601.1) twice, at least one made by urologist.	Diagnosed with ED (ICD-9-CM code 607.84) twice, at least one made by urologist	From the same database, received no diagnosis of ED	7
**Retrospective cohort study**									
**Gonen, 2005**	Turkey	CP/CPPS: 39.01, 21–55, Controls: 35, 20–49.	CP/CPPS: 66, controls: 30	Hospital based	Interview with questionnaire survey	Medical history, or microscopic examinations of prostatic fluids and urine	Self-report symptoms	Healthy volunteers with no prostatitis-like symptoms	3
**Bartoletti, 2007**	Italy	CP/CPPS: 24–43, Controls: 28–41	CP/CPPS: 764, controls: 152	Multi-hospital based	Interview with questionnaire survey	Symptoms related to CP/CPPS that were not explained otherwise	Self-report symptoms	Infertile males with normal hormonal, anatomical and functional conditions, without CP/CPPS symptoms or concomitant medications	5
**Sönmez, 2011**	Turkey	CP/CPPS: 22–48, Controls: 24–48	CP/CPPS: 43, controls: 20	Hospital based	Interview with questionnaire survey	Pain or disturbance in pelvic region for more than 3 months or medical history of CPPS, and NIH-CPSI score >14	International Index of Erectile Function	Patient without sign of urological infection and pain or disturbance in the pelvic region	5
**Mo, 2014**	China	CP/CPPS: 18–50, Controls: 18–46	CP/CPPS: 600, controls: 30	Multi-hospital based	Questionnaire survey	NIH-CPSI score ≥5 and symptoms of CP for more than 3 months.	IIEF-5 score ≤21	Volunteers with normal routine urinalysis, and a score of NIH-SCPSI ≤5	5

CP = chronic prostatitis, CPPS = chronic pelvic pain syndrome, CP/CPPS = chronic prostatitis/chronic pelvic pain syndrome, ED = erectile dysfunction, IIEF-5 = 5-item International Index of Erectile Function, NIH-CPSI = National Institutes of Health chronic prostatitis symptom index.

### Quality of included studies

The overall quality assessment scores of the included studies are listed in Table 1, and the detailed quality assessment tables are shown in Tables A and B in S1 Appendix. Four studies were community based, all of which has a cross-sectional design [22–24,31] and six were hospital based [25–30]. Three of the involved studies have no ED case in the control group [27–29]. These were retrospective cohort studies with relatively low quality scores. Most of the included case–control and retrospective cohort studies did not report adjusted OR. Two case–control studies adjusted confounding factors in the statistical analysis: Chung et al reported an adjusted OR (adjusted for monthly income, geographical location, urbanization level, hypertension, diabetes, coronary heart disease, renal disease, obesity and alcohol abuse/alcohol dependence syndrome status) without mentioning smoking history in ED cases and controls [25], whereas Elbendary et al reported insignificance of CPPS as a risk factor of ED in univariate analysis and did not report the adjusted OR [26]. Five studies included participants older than 50 years old [22–25,28], whose lower urinary tract symptoms may be mixed with CP/CPPS [32].

### Overall assessment of CP/CPPS and ED

**Fig 2:**
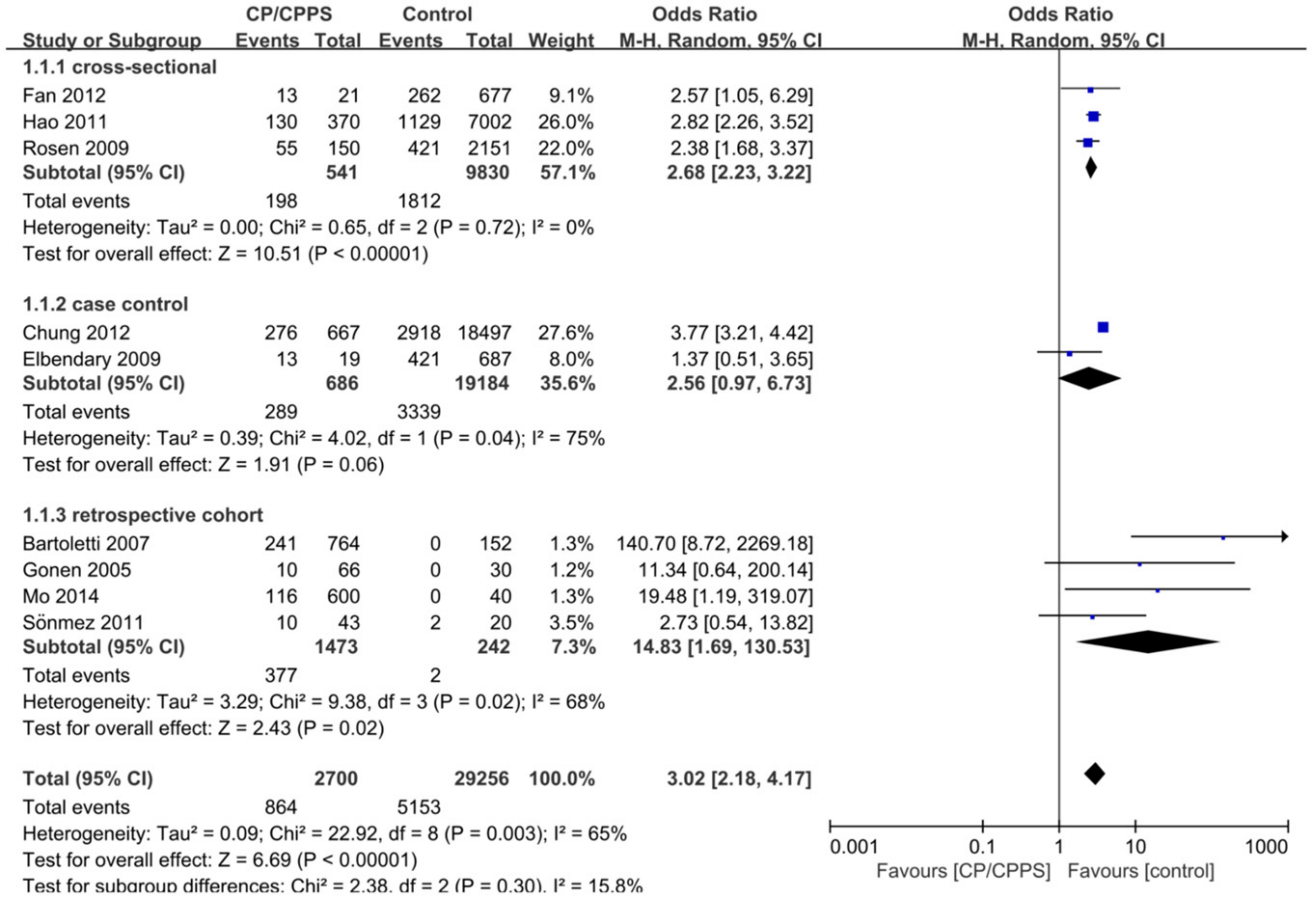
Pooled odds ratio of ED between CP/CPPPS group and control group in cross-sectional, case-control and retrospective cohort studies. CI = confidence interval, CP/CPPS = chronic prostatitis/chronic pelvic pain syndrome.

**Fig 3:**
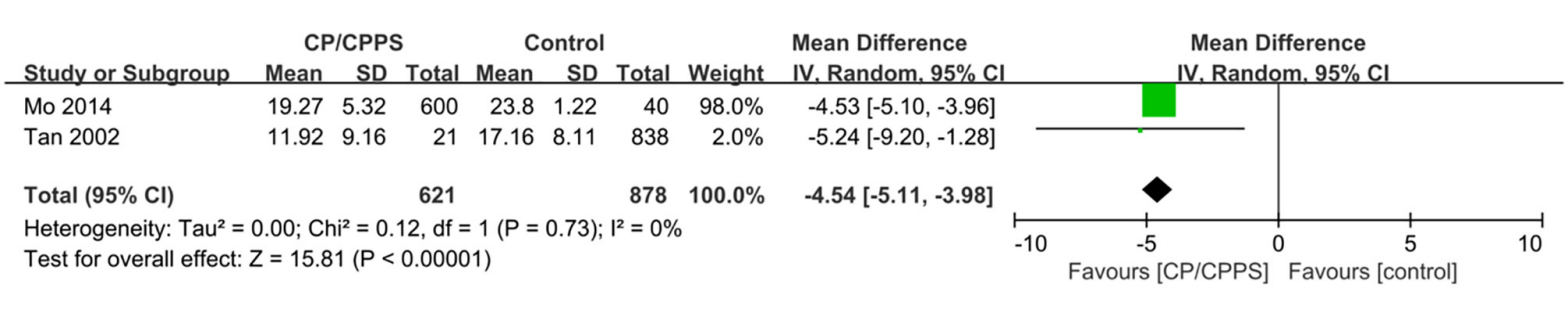
Pooled mean difference of IIEF-5 score between CP/CPPPS group and control group. CI = confidence interval, CP/CPPS = chronic prostatitis/chronic pelvic pain syndrome, IIEF-5 = 5-item International Index of Erectile Function.

Nine studies reported the number of ED cases in men with and without CP/CPPS. For the study that reported two definitions for ED and CP/CPPS [22], IIEF-5 reported ED and NIH criteria of CP/CPPS were chosen for the overall data synthesis. The forest plot is shown in Fig 2. The overall result showed a strong correlation between CP/CPPS and ED [pooled OR: 3.02, 95% confidence interval (CI): 2.18–4.17, *P* < 0.01]. However, heterogeneity across studies was significant (*I* ^2^ = 65%, *P* < 0.01) [21]. The between-study variance measured by Tau^2^ was 0.079 (Tau = 0.279, 95% CI: 0.083–2.502, calculated by R). The unadjusted OR of each study is also shown in Fig 2. Only two studies provided sufficient information for calculating a pooled mean difference in the IIEF-5 scores between the CP/CPPS and control groups. The result is shown in Fig 3. A significant decrease in the IIFE-5 score was observed in the CP/CPPS group (pooled mean difference: −4.54, 95% CI: −5.11–−3.98, *P* < 0.01). No significant heterogeneity was found (*I* ^*2*^ = 0%, *P* = 0.73).

### Subgroup analysis

We performed subgroup analysis to explore the potential source of heterogeneity. Studies were divided into subgroups in accordance with the study design, NIH type of CP, quality, territory, zero-event, ED definition, CP/CPPS definition, and age selection. The study design included cross-sectional, case–control, and cohort. Two studies reported CP in type IIIA and IIIB [29,30], one of them only involved type IIIA patients [29]. Included studies were divided into high quality and low quality on the basis of their quality assessment scores. Studies with > 5 NOS score or more than 6 “Yes” in the 11-item table were defined as high quality. The territory was divided into America (in the USA [23]), East Asia (all in China [22,24,25,29]), and Mediterranean (in Italy [27], Egypt [26], and Turkey [28,30]). Studies with and without zero-event were also reported. ED and CP/CPPS definitions were divided into self-report or medical history and scale report. For one study that reported two definitions of ED and CP/CPPS [22], we extracted data in different diagnosis criteria and synthesized these data in each subgroup. We set 50 as the age limit because men older than 50 have an increasing prevalence of benign prostatic hyperplasia (BPH), which has overlapped symptoms with CP/CPPS [32]. The results of subgroup analysis are shown in Table 2.

**Table 2:** Subgroup analyses of the association between ED and CP/CPPS.

Subgroup	No. of studies	OR(95% CI)	p value	I^2^, %	p value for heterogeneity	
					Between studies	Between subgroups
**Study design**						
**Cross-sectional**	3	2.68(2.23,3.22)	<0.001	0	0.72	0.003
**Case control**	2	2.56(0.97,6.73)	0.06	75	0.04	0.003
**Cohort**	4	14.83(1.69,130.53)	0.02	68	0.02	0.003
**Type of CP**						
**Type IIIa**	2	7.28(1.63,32.41)	0.009	0	0.34	0.21
**Type IIIb**	1	1.64(0.27,9.98)	0.59	N/A	N/A	0.21
**Population**						
**Hospital based**	6	5.00(1.82,13.68)	0.002	69	0.006	0.23
**Community based**	3	2.68(2.23,3.22)	<0.001	0	0.72	0.23
**Quality**						
**High quality**	3	2.76(1.55,4.93)	<0.001	57	0.1	0.51
**Low quality**	6	3.68(1.95,6.97)	<0.001	72	0.003	0.51
**Territory**						
**America**	1	2.38(1.68,3.37)	<0.001	N/A	N/A	0.27
**East Asia**	4	3.29(2.53,4.27)	<0.001	52	0.1	0.27
**Mediterranean**	4	7.26(0.61,86.86)	0.12	86	<0.001	0.27
**Zero event**						
**With zero event**	3	32.15(6.12,168.98)	<0.001	4	0.35	0.005
**Without zero event**	6	2.86(2.24,3.66)	<0.001	56	0.05	0.005
**ED definition**						
**Self-report**	4	5.37(3.19,9.06)	<0.001	83	<0.001	0.19
**Scale report**	6	2.64(2.21,3.16)	<0.001	0	0.49	0.19
**CP/CPPS definition**						
**Medical history or self-report symptom**	6	3.15(2.11,4.71)	<0.001	72	0.003	0.01
**NIH-CPSI score**	4	2.36(2.03,2.74)	<0.001	0	0.51	0.01
**Age selection**						
**Include age >50**	5	3.04(2.39,3.85)	<0.001	55	0.06	0.44
**Exclude age >50**	4	8.26(0.66,104.20)	0.1	87	<0.001	0.44

CP/CPPS = chronic prostatitis/chronic pelvic pain syndrome, ED = erectile dysfunction.

### Sensitivity analysis

**Fig 4:**
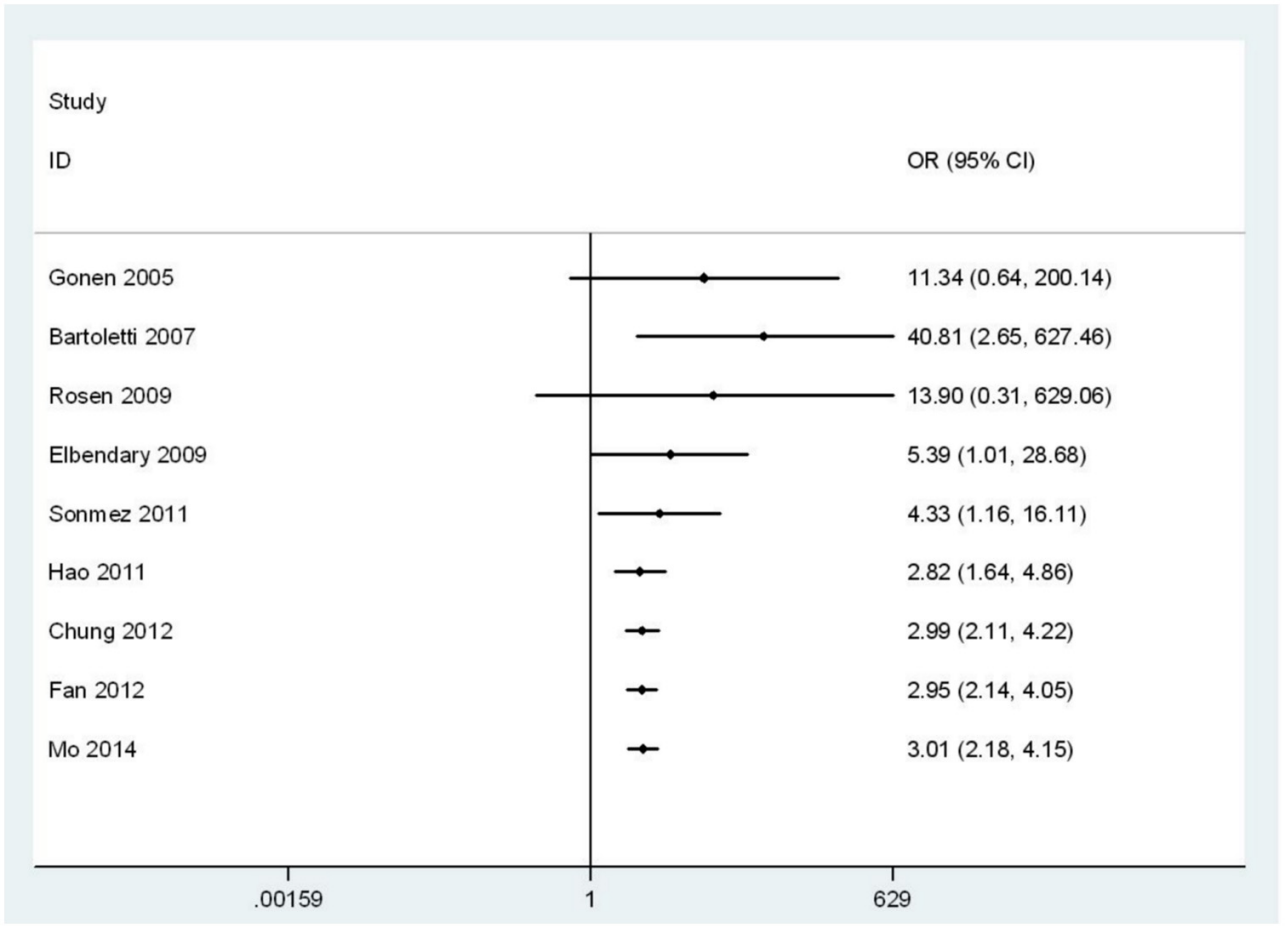
Cumulative meta-analysis of odds ratio sorted by publication year. CI = confidence interval.

We performed a meta-analysis using a fixed-effects model and found that the pooled OR did not markedly change. Omitting one study in each turn also did not affect the overall conclusion. Results of the cumulative meta-analysis of OR sorted by publication year are shown in Fig 4. After six studies were included, the pooled effect had been consistent since 2011 and the inclusion of new studies did not substantially change the result. Pooled meta-analysis based on adjusted ORs is shown in Figure A in S1 Appendix, and the conclusion was not changed.

### Publication bias

Peters’ test indicated no evidence of publication bias among studies of CP/CPPS and ED risk (*P* = 0.461).

## Discussion

Sexual dysfunction is thought to be more prevalent in CP/CPPS patients, and CP/CPPS is known to be closely related to premature ejaculation [33] and nonpremature ejaculatory dysfunctions such as painful ejaculation [34] in previous studies. As for the relationship between CP/CPPS and ED, there is still some controversy. Our meta-analysis of the existing studies indicated a significant negative effect of CP/CPPS on erectile function. Overall, a triple CP/CPPS exposure was observed in ED patients, indicating that CP/CPPS patients may have a higher risk of suffering from ED. The IIEF-5 score of patients with CP/CPPS was 4.54 point lower than that of controls. The total score for the IIEF-5 questionnaire is 25. Thus, this decrease is clinically significant, and indicates a decreased erectile function. Although the best evidence we can acquire were from case–control and retrospective cohort study, which has inevitable recall bias and selection bias, the result of the pooled meta-analysis is stable according to our sensitivity analysis. These results remind physicians to pay more attention to erectile function in patients with CP/CPPS. In accordance with our opinion, Magri et al also support that therapy for concomitant ED may improve the quality of life of patients with CP/CPPS [11]. Moreover, an Italian randomized clinical trial demonstrated that therapy for CP/CPPS can improve erectile function of ED patients [35], indicating treatment for CP/CPPS in ED patients is worth considering.

CP/CPPS may be an independent risk factor of ED, and other factors associated with CP/CPPS may also participate in this course. Inflammatory cytokines produced by prostatitis may cause inflammatory vascular disease, thereby affecting smooth muscle relaxation and endothelial function [36]. Some risk factors associated with ED also have higher prevalence in patients with CP/CPPS [14]. Furthermore psychological comorbidities are common in patients with CP/CPPS [37]. These psychological factors or comorbidities, especially depression and anxiety [29], negatively affect sexual function [38]. Most of the included studies did not report the duration of CP/CPPS, and 3 months may not be long enough to develop organic erectile dysfunction. Therefore, we infer that psychological factors may influence the genesis of ED in patients with CP/CPPS.

Of all the primary studies included, three found no statistical correlation between CP/CPPS and ED [26,28,30]. Among these three studies, one excluded patients with a history suggesting the diagnosis of psychogenic ED [26]; one had zero-event of ED in the control group and comorbid premature ejaculation in all ED cases [28]; and one used the International Index of Erectile Function, not IIEF-5, for ED diagnosis and had a small total sample size of 63 participants [30].

Substantial heterogeneity was observed across all the included studies. This result was expected because of the differences in the study design, characteristics of population, ascertainment of ED and CP/CPPS, and adjustment for other factors. Subgroup analysis showed little heterogeneity among studies with a cross-sectional design, community based population and diagnosis of ED and CP/CPPS based on scales. Studies with these properties are more representative of the population, have a decreased selection bias on controls, and utilize a more stable and exact diagnostic tool than vague and subjective symptoms.

Some studies with relatively low quality scores were included in our systematic review, and zero-event of ED occurred in the control group of these studies. Some of the zero-events might be attributed to the small sample size of the control group (30 and 40, respectively)[28,29], in consideration of the prevalence of ED [2]. In one study with 152 controls [27], zero-event might be ascribed to the selection of controls from infertility outpatients with normal hormonal, anatomical, and functional conditions. In addition, the ED diagnostic criterion, based on self-report symptoms, was not as sensitive as the IIEF-5 scale in detecting mild ED. NIH-CPSI pain score is strongly associated with prostatitis-like symptoms, while the urinary symptom and quality of life impact scores partly reflect BPH symptoms [39]. BPH might be confounded with CP/CPPS in studies applying NIH-CPSI total score for the diagnosis of CP/CPPS [30], especially for studies that included senior citizens in the cohort. ED shares risk factors with cardiovascular disease [1]; these factors include smoking, diabetes, and hyperlipidemia. Many the included studies did not adjust these factors or neglected some important confounding factors for CP/CPPS and the control group. Hence, bias was inevitable.

The present study has some limitations. The number of included studies is insufficient, and some studies have a relatively small sample size. This limitation may also lower the power of Peters’ test for detecting publication bias. Moreover, some studies did not use IIEF-5 to assess ED or did not report IIEF-5 scores. Thus, only two studies were employed to calculate the mean difference of the IIEF-5 scores, which was insufficient. Therefore, further studies on this topic should employ objective diagnostic criteria. A prospective design based on community population is preferred. Known risk factors of ED should be considered and adjusted in future studies. If possible, psychological state should be assessed.

In summary, the currently available evidence shows that patients with CP/CPPS have increased risk of ED. We suggest that the assessment of erectile function is necessary for patients with CP/CPPS. Therapy for comorbid ED may improve the quality of life of patients with CP/CPPS. Higher level of evidence from studies with a large sample size and rigorous design is needed to verify the correlation between CP/CPPS and ED.

## Supporting Information

S1 PRISMA Checklist PRISMA 2009 Checklist. (DOC)

S1 Appendix (DOC)
