# Supplementary material for: The Effect of Chronic Prostatitis/Chronic Pelvic Pain Syndrome (CP/CPPS) on Erectile Function: A Systematic Review and Meta-Analysis
Source: PLoS One. 2015 Oct 28;10(10):e0141447. doi: 10.1371/journal.pone.0141447 (PMC4625019; doi:10.1371/journal.pone.0141447)
Supplement: S1 Appendix — (DOC) [file pone.0141447.s002.doc]

**Document A. Search strategy**

PubMed

("Erectile Dysfunction"[Mesh] OR “erectile dysfunction”[Title/Abstract] OR impotence[Title/Abstract] OR “erection failure”[Title/Abstract] OR “sexual dysfunction”[Title/Abstract] or "Penile Erection"[Mesh] or “penile erection”[tiab] or "erection"[tiab] or "Sexual Dysfunction, Physiological"[Mesh] or “Sexual Dysfunctions, Psychological”[Mesh]) AND ("Prostatitis"[Mesh] OR prostatitis[Title/Abstract] OR “chronic pelvic pain”[Title/Abstract] OR “pelvic pain”[Title/Abstract] OR prostatism[Title/Abstract] OR prostatodynia[Title/Abstract] OR “abacterial prostatitis”[Title/Abstract] OR “nonbacterial prostatitis”[Title/Abstract] OR “chronic prostatitis”[Title/Abstract] or “Chronic pelvic pain syndrome”[Title/Abstract] or Prostatitides[Title/Abstract] or "Pelvic Pain"[Mesh] or "CP/CPPS"[Title/Abstract])

Embase

#1

'chronic prostatitis'/exp OR 'pelvis pain syndrome'/exp OR 'prostatitis' OR 'chronic pelvic pain' OR 'pelvic pain' OR prostatism OR prostatodynia OR 'abacterial prostatitis' OR 'nonbacterial prostatitis' OR 'chronic prostatitis' OR 'chronic pelvic pain syndrome' OR 'prostatitide' OR 'cpps' OR 'cp/cpps'

#2

'male sexual dysfunction'/exp OR 'impotence'/exp OR 'penis erection'/exp OR 'erectile dysfunction' OR impotence OR 'erection failure' OR 'sexual dysfunction' OR 'penile erection' OR 'erection'

#1 AND #2

Web of science

(TS=(“erectile dysfunction” OR impotence OR “erection failure” OR “sexual dysfunction$” or “penile erection” or "erection") or TI =(“erectile dysfunction” OR impotence OR “erection failure” OR “sexual dysfunction$” or “penile erection” or "erection")) and (TS=("Prostatitis" OR “chronic pelvic pain$” OR “pelvic pain$” OR prostatism OR prostatodynia OR “abacterial prostatitis” OR “nonbacterial prostatitis” OR “chronic prostatitis” or “Chronic pelvic pain syndrome$” or Prostatitide$ or "CPPS" or "CP/CPPS") or TI =("Prostatitis" OR “chronic pelvic pain$” OR “pelvic pain$” OR prostatism OR prostatodynia OR “abacterial prostatitis” OR “nonbacterial prostatitis” OR “chronic prostatitis” or “Chronic pelvic pain syndrome$” or Prostatitide$ or "CPPS" or "CP/CPPS"))

Cochrane Library

#1 MeSH descriptor: [Erectile Dysfunction] explode all trees

#2 MeSH descriptor: [Sexual Dysfunction, Physiological] explode all trees

#3 MeSH descriptor: [Sexual Dysfunctions, Psychological] explode all trees

#4 MeSH descriptor: [Penile Erection] explode all trees

#5 erectile dysfunction

#6 impotence

#7 erection failure

#8 sexual dysfunction

#9 penile erection

#10 erection

#11 #1 or #2 or #3 or #4 or #5 or #6 or #7 or #8 or #9 or #10

#12 MeSH descriptor: [Prostatitis] explode all trees

#13 MeSH descriptor: [Pelvic Pain] explode all trees

#14 prostatitis

#15 pelvic pain

#16 chronic pelvic pain

#17 chronic pelvic pain syndrome

#18 prostatism

#19 prostatodynia

#20 abacterial prostatitis

#21 nonbacterial prostatitis

#22 chronic prostatitis

#23 Prostatitides

#24 CPPS

#25 #12 or #13 or #14 or #15 or #16 or #17 or #18 or #19 or #20 or #21 or #22 or #23 or #24

#26 #11 and #25

**Table A. Eleven item quality assessment table for cross-sectional studies**

| Study ID | Ques.1 | Ques.2 | Ques.3 | Ques.4 | Ques.5 | Ques.6 | Ques.7 | Ques.8 | Ques.9 | Ques.10 | Ques.11 | Total score |
| --- | --- | --- | --- | --- | --- | --- | --- | --- | --- | --- | --- | --- |
| Rosen 2009 | Yes | Yes | Yes | Yes | Unclear | No | Unclear | No | Unclear | Yes | No | 5 |
| Tan 2002 | Yes | Yes | Yes | Yes | Unclear | No | Yes | Yes | Yes | Yes | No | 8 |
| Hao 2011 | Yes | Yes | Yes | Yes | Unclear | No | Yes | No | Unclear | Yes | No | 6 |
| Fan 2012 | Yes | Yes | Yes | Yes | Unclear | No | Yes | No | Yes | Yes | No | 7 |

**Table B. Newcastle-Ottawa Quality Assessment scale for case-control and cohort studies**

| Study ID | Study type | Selection | | | | Comparability | Exposure/Outcome | | | Total score |
| --- | --- | --- | --- | --- | --- | --- | --- | --- | --- | --- |
|  |  | Ques. 1 | Ques. 2 | Ques. 3 | Ques. 4 |  | Ques. 1 | Ques. 2 | Ques. 3 |  |
| Chung 2012 | case-control | * | * | - | * | * | * | * | * | 7 |
| Elbendary 2009 | case-control | * | * | - | * | * | - | * | * | 6 |
| Gonen 2005 | retrospective cohort | - | - | * | - | - | - | * | * | 3 |
| Bartoletti 2007 | retrospective cohort | * | - | * | - | * | - | * | * | 5 |
| Sönmez 2011 | retrospective cohort | * | - | * | - | * | - | * | * | 5 |
| Mo 2014 | retrospective cohort | * | - | * | - | * | - | * | * | 5 |

**Figure A. Pooled meta-analysis based on adjusted OR**


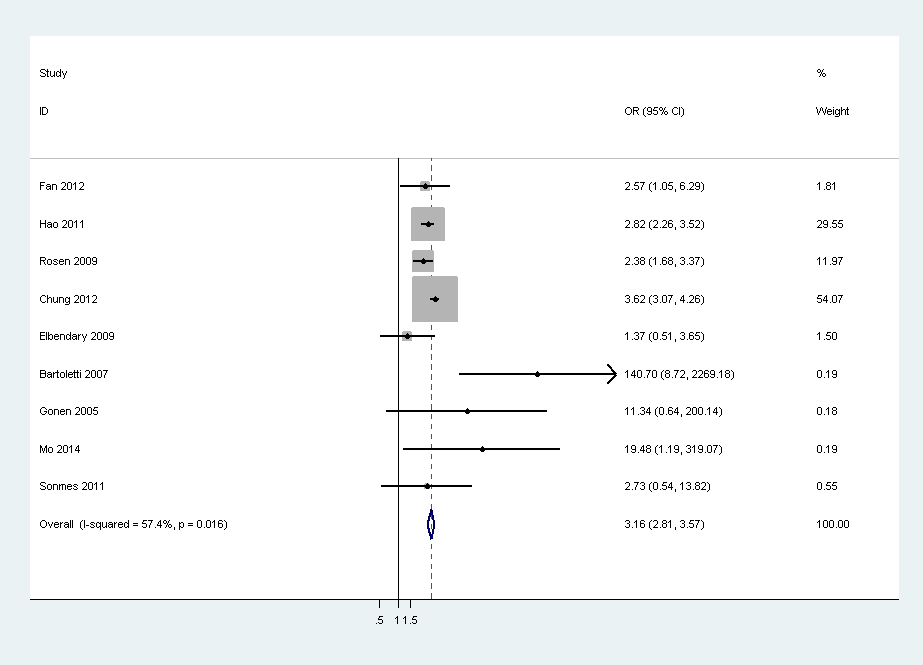


**Document B. List of full-text excluded articles**

1. Wu WC, Chung SD, Lin HC. Association between chronic prostatitis/chronic pelvic pain syndrome and erectile dysfunction: A population-based study. Journal of Urology. 2012; 187: e467-e468.

Reason for exclusion: Conference presentation with insuffiecient information and duplicated with included study (Chung, 2012).

1. Nakamura M, Fujimura T, Nagata M, Hosoda C, Suzuki M, et al. Association between lower urinary tract symptoms and sexual dysfunction assessed using the core lower urinary tract symptom score and International Index of Erectile Function-5 questionnaires. Aging Male. 2012; 15: 111-114.

Reason for exclusion: Did not define erectile dysfunction and did not report the number of participants with erectile dysfunction.

1. Aubin S, Berger RE, Heiman JR, Ciol MA. The association between sexual function, pain, and psychological adaptation of men diagnosed with chronic pelvic pain syndrome type III. J Sex Med. 2008; 5: 657-667.

Reason for exclusion: Did not define erectile dysfunction and did not report the number of participants with erectile dysfunction.

1. Lotti F, Corona G, Mancini M, Biagini C, Colpi GM, et al. The association between varicocele, premature ejaculation and prostatitis symptoms: Possible mechanisms. Journal of Sexual Medicine. 2009; 6: 418.

Reason for exclusion: Inapparopriate grouping.

1. Lotti F, Corona G, Mancini M, Biagini C, Colpi GM, et al. The association between varicocele, premature ejaculation and prostatitis symptoms: Possible mechanisms. International Journal of Andrology. 2010; 33: 84.

Reason for exclusion: Conference presentation with insuffiecient information and duplicated with reference 4.

1. Lotti F, Corona G, Mancini M, Biagini C, Colpi GM, et al. The association between varicocele, premature ejaculation and prostatitis symptoms: possible mechanisms. J Sex Med. 2009; 6: 2878-2887.

Reason for exclusion: Conference presentation with insuffiecient information and duplicated with reference 4.

1. Zhang K, He LJ, Yu W, Wang Y, Bai WJ, et al. Association of depression/anxiety with lower urinary tract symptoms and erectile dysfunction in Chinese men aged from 22 to 50 years. Beijing Da Xue Xue Bao. 2013; 45: 609-612.

Reason for exclusion: Inapparopriate grouping.

1. Lotti F, Corona G, Rastrelli G, Forti G, Jannini EA, et al. Clinical correlates of erectile dysfunction and premature ejaculation in men with couple infertility. J Sex Med. 2012; 9: 2698-2707.

Reason for exclusion: Inapparopriate grouping.

1. Lutz MC, Roberts RO, Jacobson DJ, McGree ME, Lieber MM, et al. Cross-sectional associations of urogenital pain and sexual function in a community based cohort of older men: Olmsted County, Minnesota. Journal of Urology. 2005; 174: 624-628.

Reason for exclusion: Did not define erectile dysfunction and inapparopriate grouping.

1. Burke JP, Jacobson DJ, McGree ME, Nehra A, Roberts RO, et al. Diabetes and sexual dysfunction: Results from the Olmsted County study of urinary symptoms and health status among men. Journal Of Urology. 2007; 177: 1438-1442.

Reason for exclusion: Did not define and evaluate chronic prostatitis.

1. Wang X, Cui S, Gong Z, Tang T, Gu J. The effects of chronic prostatitis/chronic pelvic pain syndromes on mental and sexual function. Chinese Journal of Andrology. 2013; 27: 41-44.

Reason for exclusion: No control group.

1. Dardavessis TH, Maravelakis PE, Asvestis C. Epidemiology of chronic prostatitis/chronic pelvic pain syndrome (CP/CPSS) and sexual dysfunction in Greek men. International Journal of Andrology. 2010; 33: 95.

Reason for exclusion: Conference presentation with insufficient data of grouping and outcome.

1. Mehik A, Hellstrom P, Sarpola A, Lukkarinen O, Jarvelin MR. Fears, sexual disturbances and personality features in men with prostatitis: a population-based cross-sectional study in Finland. BJU Int. 2001; 88: 35-38.

Reason for exclusion: Did not report number of participants with erectile dysfunction in non-prostatitis group.

1. Wein AJ, Coyne KS, Tubaro A, Sexton CC, Kopp ZS, et al. The impact of lower urinary tract symptoms on male sexual health: EpiLUTS. BJU Int. 2009; 103 Suppl 3: 33-41.

Reason for exclusion: A mixed report of prostatitis and enlarged prostate.

1. Xu X, Zhang Y, Yekeen TA, Li Y, Zhuang B, et al. Increase male genital diseases morbidity linked to informal electronic waste recycling in Guiyu, China. Environ Sci Pollut Res Int. 2014; 21: 3540-3545.

Reason for exclusion: Did not define and evaluate erectile dysfunction.

1. Permpongkosol S, Kongkakand A, Ratana-Olarn K, Tantiwong A, Tantiwongse K. Increased prevalence of erectile dysfunction (ED): results of the second epidemiological study on sexual activity and prevalence of ED in Thai males. Aging Male. 2008; 11: 128-133.

Reason for exclusion: A mixed report of prostatism/prostatitis.

1. Lan T, Wang YM, Chen Y. [Investigation of sexual dysfunction among chronic prostatitis patients in high altitude area]. Zhonghua Nan Ke Xue. 2009; 15: 886-890.

Reason for exclusion: No control group of chronic prostatitis.

1. Davis SN, Binik YM, Amsel R, Carrier S. Is a sexual dysfunction domain important for quality of life in men with urological chronic pelvic pain syndrome? Signs "UPOINT" to yes. J Urol. 2013; 189: 146-151.

Reason for exclusion: No control group of chronic prostatitis.

1. Hansen BL. Lower urinary tract symptoms (LUTS) and sexual function in both sexes. European Urology. 2004; 46: 229-234.

Reason for exclusion: Did not define and evaluate chronic prostatitis.

1. Rosen RC, Link CL, Mollon P, Aiyer LP, O'Leary MP, et al. Lower urinary tract symptoms (LUTS) and sexual health: The role of gender, lifestyle and medical comorbidities. European Urology, Supplements. 2009; 8: 171.

Reason for exclusion: Conference presentation with insuffiecient information and duplicated with included study (Rosen, 2009)

1. Rosen RC, Link CL, Mollon P, Aiyer LP, O'Leary MP, et al. Lower Urinary Tract Symptoms (LUTS) and sexual health: The role of gender, lifestyle and medical co-morbidities. Journal of Urology. 2009; 181: 161.

Reason for exclusion: Conference presentation with insuffiecient information and duplicated with included study (Rosen, 2009).

1. Giulianelli R, Pecoraro S, Sepe G, Leonardi R, Gentile BC, et al. Multicentre study on the efficacy and tolerability of an extract of Serenoa repens in patients with chronic benign prostate conditions associated with inflammation. Archivio Italiano di Urologia e Andrologia. 2012; 84: 94-98.

Reason for exclusion: No control group of chronic prostatitis.

1. Ferris JA, Pitts MK, Richters J, Simpson JM, Shelley JM, et al. National prevalence of urogenital pain and prostatitis-like symptoms in Australian men using the National Institutes of Health Chronic Prostatitis Symptoms Index. BJU International. 2010; 105: 373-379.

Reason for exclusion: Did not define and evaluate erectile dysfunction.

1. Chung SD, Chen YK, Kang JH, Keller JJ, Huang CC, et al. Population-based estimates of medical comorbidities in erectile dysfunction in a Taiwanese population. J Sex Med. 2011; 8: 3316-3324.

Reason for exclusion: Duplicated with included study (Chung, 2012).

1. Porst H, Montorsi F, Rosen RC, Gaynor L, Grupe S, et al. The Premature Ejaculation Prevalence and Attitudes (PEPA) survey: Prevalence, comorbidities, and professional help-seeking. European Urology. 2007; 51: 816-824.

Reason for exclusion: Did not define and evaluate chronic prostatitis.

1. Tang WS, Khoo EM. Prevalence and Correlates of Premature Ejaculation in a Primary Care Setting: A Preliminary Cross-Sectional Study. Journal Of Sexual Medicine. 2011; 8: 2071-2078.

Reason for exclusion: A mixed definition of prostate disease.

1. Pitts M, Ferris J, Smith A, Shelley J, Richters J. Prevalence and correlates of three types of pelvic pain in a nationally representative sample of Australian men. J Sex Med. 2008; 5: 1223-1229.

Reason for exclusion: Did not clearly define erectile dysfunction and did not report number of participants with erectile dysfunction In each group.

1. Martin SA, Haren MT, Marshall VR, Lange K, Wittert GA. Prevalence and factors associated with uncomplicated storage and voiding lower urinary tract symptoms in community-dwelling Australian men. World Journal of Urology. 2011; 29: 179-184.

Reason for exclusion: Did not define and evaluate chronic prostatitis.

1. Collins MM, O'Leary MP, Barry MJ. Prevalence of bothersome genitourinary symptoms and diagnoses in younger men on routine primary care visits. Urology. 1998; 52: 422-427.

Reason for exclusion: Inapparopriate grouping.

1. Fekete F, Rusz A, Panovics J, Romics I. Prevalence of chronic prostatitis/CP in patients with unknown origin of erectile dysfunction/ED. International Journal Of Andrology. 2005; 28: 101-101.

Reason for exclusion: Conference presentation with insufficient information and no control group.

1. Vakalopoulos I, Dimitriadis G, Varnava C, Herodotou Y, Gkotsos G, et al. Prevalence of ejaculatory disorders in urban men - results of a random-sample survey. Andrologia. 2011; 43: 327-333.

Reason for exclusion: Inapparopriate grouping.

1. Liang CZ, Hao ZY, Li HJ, Wang ZP, Xing JP, et al. Prevalence of premature ejaculation and its correlation with chronic prostatitis in Chinese men. Urology. 2010; 76: 962-966.

Reason for exclusion: Inapparopriate grouping and duplicated with included study (Hao, 2011).

1. Liang CZ, Li HJ, Wang ZP, Xing JP, Hu WL, et al. The Prevalence of Prostatitis-Like Symptoms in China. Journal of Urology. 2009; 182: 558-563.

Reason for exclusion: Inapparopriate grouping and duplicated with included study (Hao, 2011).

1. Kunishima Y, Mori M, Kitamura H, Satoh H, Tsukamoto T. Prevalence of prostatitis-like symptoms in Japanese men: Population-based study in a town in Hokkaido. International Journal of Urology. 2006; 13: 1286-1289.

Reason for exclusion: Did not define and evaluate erectile dysfunction.

1. Tang D, Zhang X, Hao Z, Zhou J, Liang C. Prevalence of prostatitis-like symptoms in outpatients with four premature ejaculation syndromes: A study in 438 men complaining of ejaculating prematurely. International Journal of Clinical and Experimental Medicine. 2014; 7: 1829-1836.

Reason for exclusion: Did not report erectile dysfunction.

1. Hedelin H, Johannisson H, Welin L. Prevalence of the chronic prostatitis/chronic pelvic pain syndrome among 40-69-year-old men residing in a temperate climate. Scand J Urol. 2013; 47: 390-392.

Reason for exclusion: Did not define and report erectile dysfunction.

1. Beutel ME, Hessel A, Schwarz R, Brahler E. Prevalence of urinary incontinence in the German population: Comorbidity, quality of life, determinants. Urologe - Ausgabe A. 2005; 44: 232-238.

Reason for exclusion: Did not define erectile dysfunction.

1. Rizzo M, Marchetti F, Travaglini F, Trinchieri A, Nickel JC. Prevalence, diagnosis and treatment of prostatitis in Italy: A prospective urology outpatient practice study. BJU International. 2003; 92: 955-959.

Reason for exclusion: Did not define and report erectile dysfunction in control group.

1. Jaspersen-Gastelum J, Rodriguez JA, Espinosa de los Monteros FJ, Beas-Sandoval L, Guzman-Esquivel J, et al. Prostatic profile, premature ejaculation, erectile function and andropause in an at-risk Mexican population. Int Urol Nephrol. 2009; 41: 303-312.

Reason for exclusion: Did not define chronic prostatitis.

1. Brookes ST, Link CL, Donovan JL, McKinlay JB. Relationship between lower urinary tract symptoms and erectile dysfunction: results from the Boston Area Community Health Survey. J Urol. 2008; 179: 250-255; discussion 255.

Reason for exclusion: Did not report numbers of participants with erectile dysfunction in each group.

1. Wagenlehner FME, Spangenberg M, Magri V, Mehik A, Hochreiter W, et al. Risk factors analysis in patients with chronic prostatitis/chronic pelvic pain syndrome (CP/CPPS) from Finland, Germany, Italy and Switzerland: Results of a multinational observational study. European Urology, Supplements. 2013; 12: e160.

Reason for exclusion: Conference presentation with insuffiecient information and no control group.

1. Martin S, Lange K, Haren MT, Taylor AW, Wittert G. Risk factors for progression or improvement of lower urinary tract symptoms in a prospective cohort of men. Journal of Urology. 2014; 191: 130-137.

Reason for exclusion: Did not define and report chronic prostatitis.

1. Smith KB, Pukall CF, Tripp DA, Nickel JC. Sexual and relationship functioning in men with chronic prostatitis/chronic pelvic pain syndrome and their partners. Arch Sex Behav. 2007; 36: 301-311.

Reason for exclusion: Did not define and report numbers of participants with erectile dysfunction.

1. Lee SWH, Liong ML, Yuen KH, Leong WS, Cheah PY, et al. Sexual dysfunction in chronic prostatitis/chronic pelvic pain syndrome: Prevalence, characteristics and impact. Journal Of Urology. 2007; 177: 31-31.

Reason for exclusion: No control group.

1. Zhou YH, Mei RB, Zhao ST, Zhang J. [Sexual function and sexual life quality of chronic prostatitis patients: a clinical investigation]. Zhonghua Nan Ke Xue. 2010; 16: 336-340.

Reason for exclusion: Did not define and report numbers of participants with erectile dysfunction.

1. Marszalek M, Wehrberger C, Hochreiter W, Temml C, Madersbacher S. Symptoms suggestive of chronic pelvic pain syndrome in an urban population: prevalence and associations with lower urinary tract symptoms and erectile function. J Urol. 2007; 177: 1815-1819.

Reason for exclusion: Inapparopriate grouping.
